# Successful pilot implementation of mailing lethal means safety devices to veterans calling the Veterans Crisis Line

**DOI:** 10.3389/fpsyt.2024.1447639

**Published:** 2024-10-30

**Authors:** Sara J. Landes, Jolie E. Bourgeois, Nyssa D. Curtis, Jennifer E. Thropp, Ethan R. Panal, Elizabeth G. Spitzer, Susan M. Jegley, MaryGrace Lauver

**Affiliations:** ^1^ Behavioral Health Quality Enhancement Research Initiative (QUERI), Central Arkansas Veterans Healthcare System, North Little Rock, AR, United States; ^2^ Department of Psychiatry, University of Arkansas for Medical Sciences, Little Rock, AR, United States; ^3^ Veterans Crisis Line, Office of Suicide Prevention, U.S. Department of Veterans Affairs (VA) Central Office, Washington, DC, United States; ^4^ Center for Healthcare Organization and Implementation Research, VA Boston Healthcare System, Boston, MA, United States

**Keywords:** suicide prevention, lethal means safety, veterans, crisis line, implementation

## Abstract

**Introduction:**

Veterans are at greater risk for suicide than non-veterans; veterans who call the Veterans Crisis Line are at even higher risk. Firearms and poisoning are among the most common methods by which people die by suicide in the United States and access to those lethal means are risk factors for suicide. The United States Department of Veterans Affairs’ Veterans Crisis Line conducted a six-month pilot to enhance lethal means safety counseling conversations by mailing lethal means safety devices (cable gun locks and/or medication takeback envelopes) to veteran callers.

**Materials and methods:**

Veterans Crisis Line responders were selected based on quality assurance ratings, received training, and passed a knowledge check prior to participating. Veterans were eligible if they were calling for themselves and had access to firearms and/or surplus medications. The pilot was assessed using operational data and qualitative interviews with responders to assess their experience, barriers and facilitators, and suggestions for improvement.

**Results:**

Responders documented 8,323 calls from 7,005 unique phone numbers; 10.8% were eligible for cable gun locks and 8.7% were eligible for medication takeback envelopes. Responders offered cable gun locks to 652 veterans and medication takeback envelopes to 522 veterans. A total of 465 cable gun locks and 567 medication takeback envelopes were mailed to 307 veterans. Operationally, there was little impact of the pilot on call handle time. Five responders participated in qualitative interviews. They reported feeling comfortable incorporating mailing devices into their work and reported that response from veterans was positive. Their most frequent suggestion for improvement was additional training.

**Discussion:**

Results demonstrate that mailing these devices to veterans was feasible and acceptable. Call handle time results show that the Veterans Crisis Line would not need additional personnel to manage changes in call handle time associated with offering devices to all veteran callers. Full implementation of this program will require updates to procedures and policies, training, documentation system changes, additional logistical support for mailing, and a plan for ongoing evaluation.

## 1 Introduction

Compared to the general non-veteran United States (U.S.) population, veterans are at elevated risk of death by suicide. The U.S. Department of Veterans Affairs (VA) Suicide Prevention Annual Report indicated that in 2021 the age- and sex-adjusted suicide rate was 72% higher for veterans than for non-veterans (1). Moreover, veterans who call the Veterans Crisis Line (VCL) are at increased risk of death by suicide with a rate higher than that of the general veteran population (2), with rates remaining high (298 per 100,000) up through a year after their call to VCL (2). For this reason, veterans who call the VCL may benefit from additional support.

Firearms and poisoning are among the most common methods by which people die by suicide in the U.S (3). Half of all suicides in the U.S. are caused by a firearm (3) and 72% of deaths by suicide among veterans involve a firearm (1). Veterans have a higher likelihood of owning a firearm that the general population; 44.9% of veterans own at least one firearm (4). One third of veterans report that at least one of their firearms is not stored safely (5). Access to a firearm is associated with increased risk of death by suicide (6) and safe storage of firearms can reduce suicide risk. In 2021, 7.8% of veteran suicide deaths were caused by poisoning, which can involve medications (1). As the third leading cause of death by suicide in veterans (1), poisoning via access to prescription medication availability is a risk factor for suicide. Both poisoning and firearms can be highly lethal means (7, 8), with attempted suicides by firearm resulting in death approximately 90% of the time (9). Given that the time between the suicide thought or decision and the suicide attempt is often less than 60 minutes (10), interventions that reduce access to lethal means may provide sufficient time for an individual to move past a crisis state.

Lethal means safety (LMS) is an evidence-based intervention that reduces suicide risk by removing or reducing access to lethal means, putting time and distance between the person and the means during periods of crisis (11–13). Use of LMS as a suicide prevention intervention was recommended by the President’s Roadmap to Empower Veterans and End a National Tragedy of Suicide (PREVENTS) task force (12), Surgeon General’s Call to Action to Implement the National Strategy for Suicide Prevention (13), and the VA/DoD Clinical Practice Guidelines (11). When access to an individual’s primary suicide method is decreased, they are unlikely to substitute it with a different method (14, 15). Lethal means safety counseling (LMSC) is a patient-centered counseling strategy in which behaviors to increase safety are promoted by aligning evidence-based recommendations with patients’ preferences and values (16). It is effective in increasing the adoption of safety practices surrounding lethal means, reducing risk for suicide (17) and is recommended when working with individuals at risk (13). Providing LMSC can be limited by several barriers. For instance, LMSC typically occurs between a clinical provider and patient, thereby limiting its potential recipients (e.g., people currently accessing care). LMSC can include offering safe storage devices such as cable gun locks. In a recent systematic review, ten of the 14 studies that reported improved storage behavior offered a safe storage device with LMSC (18), suggesting that offering such devices may improve effectiveness of the intervention. In addition, in a recent qualitative study, veterans noted that being offered no-cost safe storage devices would motivate them to secure firearms and medications (19). Although there is strong data suggesting that LMSC can improve safe storage of firearms and medications, there are minimal studies assessing the effect of LMSC on reducing suicidal behaviors. Few studies assess changes in suicidal behavior following LMSC, suggesting the need for more research in this area (18, 20–22).

Given that the VCL is available to all veterans regardless of VA care use and/or eligibility, it offers an opportunity to reach both VA and non-VA using veterans with a critical suicide prevention intervention. In June 2022, the VCL expanded its crisis intervention work by implementing a six-month LMS pilot to mail cable gun locks and/or medication takeback envelopes, as appropriate, to veteran callers as an extension of responders’ regular LMSC. The cable gun locks, which had VCL’s logo and phone number printed on it, can be used with most firearms to prevent them from being used. Medication takeback envelopes are used to mail unused or surplus medications for anonymous destruction. This paper describes the implementation of the pilot, feasibility, operational impact on VCL, acceptability, and perspectives of participating responders.

## 2 Materials and methods

### 2.1 Overview

The operational impact of the pilot was monitored, and a qualitative evaluation was conducted to assess feasibility and acceptability. Operational impact analyses focused on veteran reach, call duration, and after call work time. Qualitative interviews were conducted with participating responders. This program evaluation project was reviewed by the authorized program office (VA’s Office of Suicide Prevention) and met criteria for classification as non-research, per VA policy (23). In addition, the local institutional review board reviewed the project and determined it was non-research.

### 2.2 Setting

The VCL is a free, 24-hour national crisis resource for veterans, service members, and third parties with concerns about veterans or service members. Of note, only veterans were included in the pilot. The VCL is staffed with highly trained responders who decrease veteran’s distress and/or suicidal ideation (24), connect veterans with appropriate resources, and coordinate care with approximately 170 VA medical centers in the U.S. As of April 2024, VCL has answered over 7.7 million calls, 377,000 text messages, and over 941,000 chats, and has made more than 1.5 million referrals to VA suicide prevention coordinators (25). The VCL is available to any veteran regardless of eligibility for VA health care and is poised to benefit a population of veterans who have historically been difficult to reach (e.g., veterans not eligible for VA health care, veterans not enrolled in VA health care).

Per VCL policy (26), risk mitigation planning and LMSC is required in situations in which veterans endorse one or more of the following: (a) current or past suicidal ideation, (b) past suicide attempts, (c) current thoughts of self-harm, or (d) current thoughts of violence toward someone else. When one or more of these conditions is met, responders are required to ask about access to means. When veterans endorse access to means, responders collaborate with them to ensure their safety.

### 2.3 Intervention

As described above, LMSC is the conversation about engaging in LMS and is already standard practice at VCL. LMS devices are items that support engaging in LMS, such as cable gun locks and medication takeback envelopes. LMSC can occur without the provision of LMS devices, but their provision can enhance the intervention (18, 27–29). This pilot evaluated the addition of providing LMS devices to LMSC.

Standard procedures were followed for all calls taken as part of the pilot. The only change was that responders would offer to mail cable gun locks and/or medication takeback envelopes to eligible veterans, as appropriate. They were encouraged to discuss with veterans how many of each item would be needed and to accommodate each veteran’s requested quantity, based on their access to firearms and/or excess medications. Post-call documentation was slightly modified to accommodate the change.

**Figure 1:**
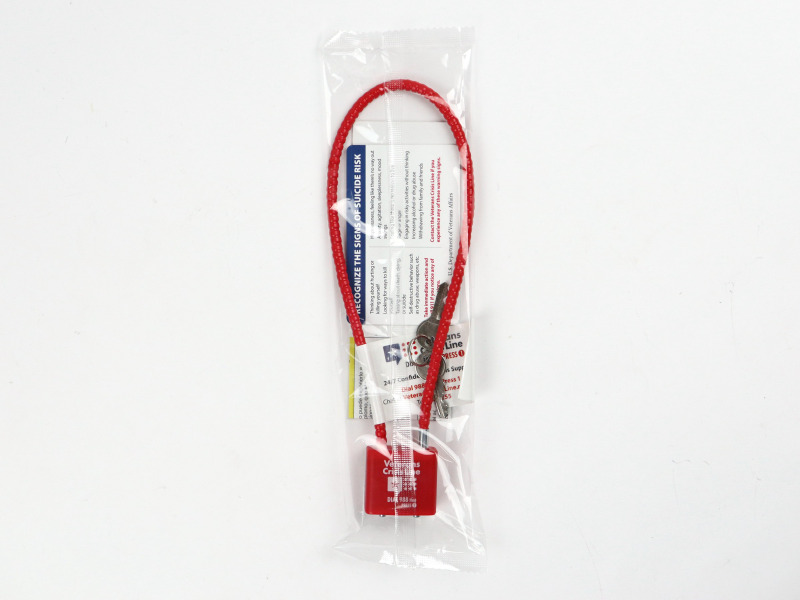
Photo of cable gun lock in packaging with wallet card and instructions.

**Figure 2:**
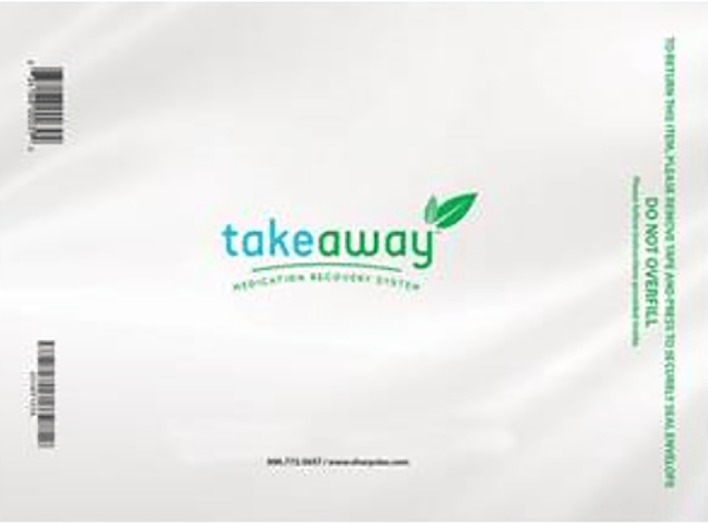
Image of a medication takeback envelope.

The cable gun locks were those already widely used by the VA, such as for distribution at local VA medical centers. They can be used with most firearms. Their packaging included instructions for use and VCL contact information on a wallet card. The lock had VCL’s logo and phone number printed on it. See **Figure 1**. The medication takeback envelopes were 8 x 11-inch envelopes with pre-printed barcodes unique to the VCL for tracking purposes. See **Figure 2**. Envelopes were provided by the National VA Pharmacy. Veterans place unused and/or surplus medications inside them and mail them back via the United States Postal Service for anonymous destruction by a Drug Enforcement Agency (DEA) Reverse Distributor. Patient identifiers and the type or dosage of medication returned are not tracked or reported. The National VA Pharmacy and the DEA Reverse Distributor use the bar codes to track the aggregate number and weight of returned envelopes.

When a veteran accepted LMS devices, responders documented their name, mailing address, and number of each device requested in VCL’s primary system of record. VCL Logistics received a weekly report for mailing. Because the intervention was intended to supplement existing VCL crisis intervention procedures and to support future risk mitigation, not to be an immediate suicide prevention measure, weekly mailings (as opposed to more frequent mailings) were not determined to be a risk. Weekly mailings were also more feasible for the logistics personnel. Logistics personnel addressed plain bubble mailers by hand; no return address was written.

### 2.4 Participants

Participants included responders and veterans calling the VCL. Pilot leads sought to have a cohort of approximately 50 responders. Responder eligibility to participate was based on established proficiency in LMSC, as determined by cumulative quality assurance (referred to as Silent Monitoring) ratings between December 2020 and March 2022. VCL’s Policy for VCL Social Science Interaction Standards and Silent Monitoring (26) consists of over 40 unique elements pertaining to responders’ call and documentation. Each element receives a “Successful,” “Not successful,” or “Not applicable” rating by Silent Monitors handling each review. Pilot leads identified six LMS-related elements within these standards and identified the top performing responders during that time frame. These six elements included 1) conducting lethal means safety counseling, 2) assessing suicidal capability: past history and current means, 3) assessing substance use capability: overdose risk potential, 4) assessing capability for violent behavior, 5) collaboratively creating a risk mitigation plan, and 6) documentation of risk mitigation plan.

#### 2.4.1 Responder eligibility, recruitment, and training

Pilot staff were approved to recruit approximately 50 VCL responders for the pilot; additionally, their goal was to recruit responders who were already skilled in LMS counseling. Therefore, responders were recruited and trained in two waves. The first wave of recruitment occurred in March 2022. Initially, the top 20% performing responders on the 6 LMS-related elements were invited to participate. All from this tier who volunteered to participate in response to the invitation were included. However, because fewer than 50 responders volunteered from this group, pilot staff then invited the top 49% according to the same criteria to participate. Because the number of volunteers was then higher than the approved cohort of 50, pilot staff used seniority level, as determined by Service Computation Date (SCD), to select interested responders from this second tier of responders. Due to attrition (three expressed interest but never completed training, and 12 withdrew from participating during the pilot), a second wave of recruitment occurred in July 2022. Responders in this wave were from the original top 49% tier and had initially volunteered to participate but were not initially selected because of the limited approved cohort size. Pilot leads again used SCD as a criterion when selecting from remaining applications. To participate in the pilot, responders were required to obtain supervisory approval.

Training for cohort 1 occurred in May 2022 and training for cohort 2 occurred in August 2022. Responders attended a 2-hour live group training session that included information about LMS in general, including relevant evidence in the literature about LMS, strategies for discussing LMS with veterans, and specific logistical procedures associated with this pilot. Because VCL staff are now almost entirely remote workers, this live training was held virtually. Responders also attended a 2-hour asynchronous, self-paced training, during which they reviewed additional LMS-related educational materials on their computers. All training took place outside of responders’ normal tours of duty, and responders were compensated with overtime pay or compensatory time off.

Participating responders completed a 12-item knowledge assessment before and after completing the asynchronous individual training session. The assessment consisted of multiple choice and true/false questions, and all responders passed with 80% correct or better before being cleared to participate in the pilot. Responders participated in the pilot during their regular tour of duty.

#### 2.4.2 Veteran eligibility

Initially, there were three eligibility criteria to receive LMS devices: callers had to (1) be a veteran calling for themselves, (2) report access to firearms and/or surplus medications, and (3) endorse one or more of the following: (a) suicidal ideation currently or in the past, (b) past suicide attempts, (d) current self-harm, or (d) current thoughts of violence. These are the situations that require LMSC, per VCL Standard Operating Procedures. However, given that all veterans are at increased risk for suicide regardless of their expressed level of crisis during the call to VCL (2), the third eligibility criterion was eliminated during the fourth month of the pilot at the request of VCL leadership.

### 2.5 Measures

Three sources of quantitative data were used to monitor the pilot. First, operational data including call duration and amount of time taken to document calls (referred to as After Call Work, or ACW), was collected through VCL’s phone system. Second, responders used VCL’s system of record to document all calls according to standard policies and pilot-specific information (e.g., mailing address, number of each type of device accepted). Third, a web-based InfoPath form was developed for the pilot to allow responders to document whether veterans were eligible for each device, whether devices were offered, and what barriers interfered with veterans’ acceptance. The InfoPath form captured information via a mixture of fields with drop-down choices and free-text responses.

A qualitative evaluation was conducted with participating responders by an external team as part of a rapid response team evaluation that requires the evaluations to be brief and time limited. Pilot responders were recruited through email and invited to participate in a qualitative interview. The first five responses were selected. The interview guide consisted of open-ended questions that focused on responder experience and comfort level offering LMSC and devices, veterans’ reactions, barriers and facilitators to implementation, and suggestions for improvements. Interviews were conducted by a psychologist with expertise in LMSC (ES). Interviews were audio recorded and transcribed verbatim.

Interview transcripts were analyzed using template analysis, a data reduction technique developed for health services research (30). An evaluation team member with qualitative experience (NC) analyzed the transcripts and created individual templates (summary tables in Word) using content analysis. Each template contained deductive domains informed by the goals of the evaluation (i.e., experience and comfort level offering LMS devices, veterans’ reactions, barriers and facilitators to implementation, and suggestions for changes to the pilot). All templates were audited by other team members for quality control and to ensure validity and consistency of results. No discrepancies were identified. The team member then synthesized the individual template data into a participant by domain matrix display, which was used to compare and identify the full range of responses in each domain.

## 3 Results

A total of 51 responders were recruited to participate. Of those, 48 completed training, and 46 worked at least one shift. Twelve responders withdrew from participating. Reasons for withdrawal included: accepting new positions (N=8), wanting to focus on honing other skills (N=2), health problems (N=1), and being removed from independent duty by a supervisor for retraining (N=1). At the end of December 2022, VCL employed 1,051 responders. Pilot responders comprised a maximum of 4.4% of them.

### 3.1 Reach

Pilot responders documented 8,323 calls from 7,005 unique phone numbers during the 6-month pilot. Responders were asked not to participate in the pilot when engaged in extra duties (e.g., training new responders) or when working in VCL’s online chat and text services. As a result, and due to attrition, the number of calls taken as part of the pilot decreased steadily.

Of the 8,323 calls taken during the pilot, responders documented that 898 (10.8% of calls) were eligible for cable gun locks, and 721 (8.7% of calls) were eligible for medication takeback envelopes. See **Table 1** for the number of calls taken during the pilot and the number and percentage of those callers who were eligible, offered, and accepted each LMS device. Responders were initially incorrectly marking veterans as eligible for the pilot when they were experiencing mental health crisis but had no access to firearms and/or surplus medications. As a result, the wording on the InfoPath form was changed about one month into the pilot to emphasize that access to means was an integral consideration when determining eligibility. Clarifying communications were disseminated via email. Excluding the first month, total eligibility drops to 8.3% and 4.8% of all calls for cable gun locks and medication takeback envelopes, respectively.

**Table 1:** Number of calls taken by pilot responders and number and percentage of those eligible for, offered, and accepted LMS devices.

		Cable gun locks			Medication takeback envelopes		
Month	# of calls N	Eligible N (%)	Offered N (% of all calls)	Accepted N (% of those offered)	Eligible N (%)	Offered N (% of all calls)	Accepted N (% of those offered)
Month 1	2,229	393 (17.6%)	227 (10.2%)	49 (21.6%)	426 (19.1%)	276 (12.4%)	68 (24.6%)
Month 2	1,738	141 (8.1%)	123 (7.1%)	28 (22.8%)	108 (6.2%)	86 (4.9%)	38 (44.2%)
Month 3	1,554	139 (8.9%)	119 (7.7%)	28 (23.5%)	75 (4.8%)	63 (4.1%)	24 (38.1%)
Month 4	1,110	99 (8.9%)	82 (7.4%)	23 (28.0%)	39 (3.5%)	34 (3.1%)	17 (50.0%)
Month 5	977	67 (6.9%)	50 (5.1%)	11 (22.0%)	44 (4.5%)	38 (3.9%)	17 (44.7%)
Month 6	715	59 (8.3%)	51 (7.1%)	23 (45.1%)	29 (4.1%)	25 (3.5%)	15 (60.0%)
Total	8,323	898 (10.8%)	652 (7.8%)	162 (24.8%)	721 (8.7%)	522 (6.3%)	179 (34.3%)

Pilot responders offered cable gun locks to 652 veterans (585, or 65.1%, of those were eligible, plus an additional 67 who were not eligible) and medication takeback envelopes to 522 veterans (481, or 66.7% of those who were eligible, plus an additional 41 who were ineligible). While the practice of offering devices to ineligible veterans diminished significantly, it persisted in lower levels throughout the pilot.

There were 341 callers who accepted one or both devices; this represented 307 unique veterans. Veterans who accepted cable gun locks accepted an average of 2.89 locks each (SD = 3.05), ranging from 1 to 30 per person, for a total of 465 cable gun locks. Those who accepted medication takeback envelopes accepted an average of 3.15 envelopes each (SD = 3.19), ranging from 1 to 20 per person, for a total of 567. See **Table 2** for the most common reasons for declining. Not all calls with eligible veterans led to the offer of LMS devices. Reasons documented included: (1) responder forgot, (2) responder was managing an imminent crisis and it was inappropriate to offer, (3) veteran disconnected the call, (4) veteran was experiencing mental health symptoms that made it impossible to have such a conversation (e.g., confusion), and (5) veteran did not have or want to share a mailing address.

**Table 2:** Reasons for declining LMS devices when offered.

Reason for declining	Cable gun lock N (%)	Medication takeback envelope N (%)
Already securely stored	220 (48.4%)	64 (20.1%)
Don’t want/wouldn’t use	106 (23.3%)	120 (37.7%)
Have a way to store already but is not currently using it	43 (9.5%)	27 (8.5%)
Did not provide a reason	18 (4.0%)	37 (11.6%)
Other	68 (15.0%)	70 (22.0%)

*Note: Reasons were not recorded for all who declined, and the InfoPath form only allowed one reason to be selected.

### 3.2 Operational metrics and forecasting

We examined all VCL calls in which responder groups, pilot and non-pilot, documented that the caller had access to lethal means (estimated at 12.33% of VCL call volume). Due to 29.67% (113,888/383,868) of all calls having extremely short talk times (i.e., < 3 minutes) which possibly precluded a complete assessment of the caller, two versions of the group comparison were performed: 1) all calls (total n = 383,868: non-pilot n = 376,748 and pilot n = 7,120) and 2) calls with talk times of at least 3 minutes (total n = 269,980: non-pilot n = 264,547 and pilot n = 5,433). In other words, while responders could determine that callers had access to lethal means during very short calls (under 3 minutes), we presume that a thorough conversation with complete LMSC would not have been possible during such calls and therefore performed analyses with both the full sample and a sample only including calls longer than 3 minutes based on this presumption. Using independent-samples t-tests, we tested for differences between groups in talk time, after-call work (ACW) time, and average speed of answer (ASA) time.

Independent two-samples tests using all call data indicated that on average pilot calls were answered 1.22 seconds faster (ASA), their talk time lasted 2.16 more minutes, and they required 0.52 fewer minutes of ACW time than non-pilot calls. While all comparisons were statistically significant (p<.0001) the effect size for each was rated very small (i.e., Cohen’s d < 0.19). See **Table 3**. These results show that the pilot protocol was associated with an average net increase of 1.64 minutes of total handle time per call (talk time + ACW time) for the group based on all calls, regardless of duration. Based on a weekly average of 1,895 LMS-related calls weekly, implementing the LMS protocol would result in an additional 3,107.8 minutes of work per week, on average or 51.80 hours per week (approximately 2,693 hours per year). This would translate roughly to an additional 2-3 full time equivalent employees (FTEE).

**Table 3:** Pilot and non-pilot comparisons all call data.

All Calls (n=383,868)	Measure	Pilot Group (n=) Mean (SD)	Non-Pilot Group (n=) Mean (SD)	Independent t-test	Cohen’s d
All Calls	ASA	8.07 seconds (15.83)	9.29 seconds (15.23)	*t*(383,866) = -6.71, *p* <.0001	-.079
All Calls	Talk Time	18.37 minutes (9.68)	16.21 minutes (19.57)	*t*(383,866) = 9.20, *p* <.0001	.110
All Calls	ACW	12.68 minutes (11.23)	13.20 minutes (12.64)	*t*(383,866) = -3.47, *p* <.0001	-.044

ACW, after call work (in minutes); ASA, average speed of answer (in seconds).

For the second version of the analysis (group comprising of a random selection of 20% of calls having talk times of at least 3 minutes), we removed 113,888 calls with less than 3 minutes of talk time from the non-null (talk time = 0 seconds) calls, leaving 269,980 calls (5,433 pilot and 264,547 non-pilot). Among those calls with a talk time of at least three minutes, a random selection of 20% of those calls was used to address statistical overpowering due to excessively large sample size. The random 20% of calls was taken from each group using the MS Excel random number generator function, resulting in 1,086 pilot calls and 52,908 non-pilot calls. In this analysis, on average pilot calls had 1.23 more minutes of talk time and 1.19 fewer minutes of ACW time than non-pilot calls. See **Table 4**. Both differences, talk time and ACW time, were significant. There was no statistically significant difference in ASA time between pilot (M = 8.73 sec., SD = 13.26 sec.) and non-pilot calls. In this sample, the pilot LMS protocol was associated with an average net increase of 0.04 min. of total time per call (talk time + ACW time). Based on a weekly average of 1,895 LMS-related calls, implementing the LMS protocol would result in an additional 75.8 minutes of work per week, on average, or 1.26 hours per week (approximately 66 hours per year). With this analysis, it appears that deploying the LMS protocol would have a low impact on operations and would not require additional FTEE.

**Table 4:** Pilot and non-pilot comparisons calls over 3 minutes data.

Calls Over 3 minutes (n=52,909)	Measure	Pilot Group (n=) Mean (SD)	Non-Pilot Group (n=) Mean (SD)	Independent t-test	Cohen’s d
All Calls	ASA	8.73 seconds (13.26)	9.24 seconds (15.25)	*t*(53,992) = -1.10, *p* = .27	
All Calls	Talk Time	23.86 minutes (20.43)	22.62 minutes (20.19)	*t*(53,992) = 1.993, *p* = .046	.067
All Calls	ACW	14.76 minutes (11.86)	15.95 minutes (13.26)	*t*(53,992) = -2.93, *p* <.0034	-.095

ACW, after call work (in minutes); ASA, average speed of answer (in seconds).

### 3.3 Qualitative evaluation

Five pilot responders participated in qualitative interviews, which lasted between 22 and 33 minutes. Four participants worked the day shift, and one worked the night shift. All displayed a high level of enthusiasm and motivation when talking about the program.

All participants reported feeling very comfortable incorporating this pilot into their work because it felt familiar and not out of the ordinary to offer LMS devices when having LMS discussions. They reported that their work experience and training they received prior to the pilot’s implementation was essential to building this level of comfort. For example, one participant stated, “I was pretty prepared because we already do the lethal means safety plans. So, offering cable gun locks and the medication takeback envelopes was just an added security, an added safety measure for the veterans.” Many stated that they use the LMS discussion as an extension of a veteran’s safety plan and that offering LMS devices provides a critical element in the safety plan that was missing before. Several participants noted that awareness of the importance of language when discussing LMS with veterans was important to their preparedness for this program. Participants stated that they are very conscientious in how they bring up the LMS discussion to not offend, judge, or cause defensiveness. They discussed using skills to evoke the desire and motivation to build safe habits and collaborate with the veteran, who then has the autonomy to decide what will work best for them.

Participants noted that veteran reactions to being offered cable gun locks and/or medication takeback envelopes were mostly positive. They stated that veterans seemed surprised and appreciative after being offered LMS devices. In one example, a participant discussed a conversation with an initially defensive veteran. They said, “I had a veteran who at first did not want to answer if he had any firearms, and then through the course of the conversation about how we are offering cable gun locks, he ended up taking ten cable gun locks. So, he went from not wanting to say that he had firearms at all to actually providing a list of what kinds of firearms he has”.

A reoccurring topic in all interviews was the perceived difference in acceptance rates between the LMS devices. Participants thought veterans accepted the cable gun locks more frequently than the medication takeback envelopes. Participants believed that veterans are more willing to accept cable gun locks because it is a tangible object they can use, which makes it easier to understand the benefit of using it. Furthermore, they believed that it gives them a sense that they are doing something good for themselves and their families. Participants noted that some veterans asked if they could have some extra gun locks to give to their veteran peers. See **Table 5** for examples of reasons veterans declined both types of devices, as reported by VCL responders participating in qualitative interviews.

**Table 5:** Examples of veteran reasons for declining each type of LMS device, as reported by VCL responders.

Cable Gun Locks	Medication Takeback Envelopes
Already own a gun safe or a lock box	Did not think their medication was a safety concern
Already received one from their local VA mental health provider	Afraid to part with their medication due to fears that they may need it in the future and would not be able to receive a refill
Discomfort providing their address due to fear that someone would call emergency responders to their home	Concern someone would steal the medication in the mail and gaining access to potentially lethal medication and their private information listed on the bottles
Belief that putting a lock on their weapon was too inconvenient for home security purposes	Worry about liability if something unfortunate happened to someone who used the medication inappropriately

Training was the most frequent recommendation mentioned for improving the pilot. While they felt the training that they received prior to the pilot’s implementation was excellent, they stated it would be beneficial to have refresher courses to maintain their proficiency and ensure they are using the most updated methods in their calls with veterans and subsequent documentation. Participants wanted to learn new ways to encourage veterans to accept and use medication takeback envelopes. Additionally, they reported wanting more specialized trainings focused on key elements of the program, how to handle uncomfortable conversations, and role-playing practice sessions using real world vignettes. Participants mentioned wanting regular team meetings with their peers and those with more experience to discuss cases, gather feedback, and generate new ideas from others. Regarding expanding the pilot, one participant suggested also offering cable gun locks and medication takeback envelopes through text message VCL services.

## 4 Discussion

To our knowledge, this was the first implementation of mailing LMS devices in a call center environment. Results demonstrate that mailing LMS devices to veterans following a call to the VCL was feasible and acceptable. VCL was able to establish a partnership with National Pharmacy that allowed them to obtain envelopes. Cable gun locks were easily obtained from VCL overstock. If needed, more could have been obtained from the same supplier that VCL originally used. Logistics personnel experienced no significant barriers to sending out mailings on a weekly basis. They were able to secure storage space for the materials, access it readily, and mail all packages as needed.

In general, it was feasible to identify responders who were eligible and willing to participate in the pilot. In terms of veteran eligibility, while we did report lower eligibility than the literature may have predicted (10.8% eligible for locks and 8.7% eligible for envelopes), this was not entirely unexpected. First, as indicated, original criteria for eligibility included the requirement that the veteran disclose current or past suicidal ideation, past suicide attempts, current self-harm, or current thoughts of violence. This criterion was not dropped until the pilot was in its fourth month. Additionally, it is also possible that veterans were reluctant to disclose to the VCL responder that they had access to firearms and/or excess medications, for fear of judgment or having them taken away. Most eligible veterans were offered cable gun locks and medication takeback envelopes as appropriate.

Operationally, there was little impact of the pilot on call handle time. Already skilled in handling LMSC, pilot responders were able to offer a LMS device without adding significant time. With the all-VCL sample comparison, it took 1.23 more minutes, and for the sample excluding calls under 3 minutes just 2.16 more minutes, to incorporate offering LMS devices into their usual call flow and documentation time, as compared to non-pilot responders. Counterintuitively, documentation time was 1.19 minutes shorter for pilot responders with the all-VCL sample comparison and 0.52 minutes shorter for pilot responders with the sample excluding calls under 3 minutes comparison, potentially due to selection of high-performing responders in LMS-related elements. This was despite the additional documentation during the pilot that non-pilot responders did not have to complete. The already-negligible ACW time could potentially have been shorter without this. As mentioned before, analysis of the all-VCL sample indicated that VCL would need 2-3 additional FTEE to manage the additional time associated with the LMS device conversation and documentation process, while analysis of the sample excluding calls under 3 minutes indicated that no additional FTEE would be needed. We expect that given sufficient time to learn and master the changes in procedure and documentation, VCL would not need additional FTEE to manage changes in handle time associated with offering LMS devices to all veteran callers. It is important to note that the method of selecting responders to participate in the pilot was based on preexisting characteristics, particularly higher quality assurance ratings, and not random selection. This preexisting higher performance level among pilot responders may partially explain the shorter mean ACW time for LMS calls relative to that of the non-LMS calls. In other words, the results may be partially influenced by responder characteristics as opposed to solely the effect of pilot activities. It is not expected that VCL would require additional FTEE to manage the logistical portion of the LMS intervention (addressing, filling, and mailing packages to veterans) because VCL would contract with a vendor for this portion.

Mailing LMS devices was acceptable to responders. Responders who participated in qualitative interviews were comfortable incorporating mailing devices into their work and they identified their previous experience and training received as being critical. Over the course of the pilot, 24.8% and 34.3% of veterans offered cable gun locks and medication takeback envelopes respectively accepted them, indicating that this is acceptable to veterans. Responders reported that veterans were appreciative of the offer to mail devices.

Differences in reasons for declining the interventions provide some insight into veterans’ perceptions about the relative risk associated with firearms and medications. A much greater percentage of veterans declined LMS devices because firearms were already stored securely (48.4%) than those who declined medication envelopes because their medications were stored securely (20.1%; see **Table 2**). Alternatively, the most frequently endorsed reason to decline medication takeback envelopes, “don’t want/wouldn’t use” (37.7%), as compared to 23.3% for cable gun locks, indicated that surplus medications were not widely considered a threat or harmful among veteran callers. One reason for declining LMS devices for firearms was “belief that putting a lock on their weapon was too inconvenient for home security purposes.” This is consistent with prior literature suggesting that veterans who believe that firearms are not useful for personal protection if the owner has to take time to load or unlock them are also less likely to safely store their firearms (5). In addition, one reason for declining medication takeback envelopes was that veterans “did not think their medication was a safety concern.” Both of these reasons suggest that there is a need for increased education around the safety concerns of firearms and medications that are not stored safely in the home. Future studies should examine ways to include information about safety in a manner that is acceptable and persuasive to veterans.

### 4.1 Implications

This pilot demonstrated that it is feasible and acceptable to mail LMS devices to eligible individuals calling the VCL, at least on a small scale. Based on this pilot and in support of the 2024 National Strategy for Suicide Prevention (31), VCL intends to do a full-scale implementation of mailing LMS devices to eligible veteran callers. This means that all VCL responders will routinely offer cable gun locks and medication takeback envelopes, as appropriate, to veteran callers during their calls. To fully implement this program, several operational changes will be needed. First, VCL will need to update relevant Standard Operating Procedures and policies. Second, VCL will need to train all staff, including responders, supervisors, and Quality Assurance staff, in the new procedures. Training may include training responders on the importance safety messages and messengers have on veteran acceptance and adoption of the LMS devices (32, 33). Third, VCL will need to update its system of record with the appropriate fields so that the process is streamlined and automated to the extent possible. Fourth, VCL will need additional logistical support to include ongoing sourcing of LMS materials and managing the logistics of ongoing mailings of materials, particularly considering continuous growth in overall call volume, though contracting with a vendor for mailing the LMS devices would mitigate the risk of needing additional FTEE for this. Finally, VCL will need to plan for ongoing evaluation of LMS-related data to monitor the operational impact of new activities, reach of the devices, and effects on the veterans sent the devices.

### 4.2 Limitations

This project was not without limitations. Importantly, the size of the responder cohort was small; 46 responders participated in the pilot for at least one shift. Additionally, as an effort was made to recruit responders already skilled in LMSC, it is possible that this cohort is not fully representative of all VCL responders. The number of responders who were recruited for the qualitative evaluation was small given the mechanism used for that component of the evaluation. However, no new themes were identified after five interviews given the practical nature of the interview. Also, the percentage of calls with veterans eligible for LMS devices was quite small. Therefore, while the response that we observed from veterans was overwhelmingly positive, it was a small group and may not be generalizable to all VCL callers or callers to other crisis hotlines. Another limitation is that the pilot was limited to calls to the VCL and not offered to veterans contacting VCL via text message and online chat. While chats are anonymous and would prohibit offering of LMS devices, texts are treated similarly to calls and would allow for offering of LMS devices. Limiting the pilot to calls allowed for evaluation of initial feasibility and acceptability but did not allow for gathering of similar information as it relates to interactions with veterans via text message. Additionally, this pilot did not include demographic information about the responders, including if they were veterans or firearm owners and thus, may be viewed as peers to VCL callers. Previous literature has suggested that veterans perceive military personnel as more credible messengers about LMS (32) and that peer messengers may influence the veterans accepting LMS devices (34). Future studies should include how demographics of the responders, such as their veteran status, may influence acceptability of LMS.

### 4.3 Conclusion

While it is established that providing LMSC to individuals at high risk for suicide has a preventative effect in general (11–13), it was not known how well this concept would apply to the VCL, a crisis call center that manages telephonic and virtual crisis interactions with veterans at especially high risk for suicide. Implementing a pilot in which a small cohort of responders offered to mail LMS devices to veterans had little, if any, risk, and it appeared to provide evidence that the changed procedure was appreciated by both responders and the veterans with whom they interacted. It also did not create a noticeable burden on VCL operations. Long-term implications regarding suicide risk and suicidal behaviors among the veterans who received the devices are yet unknown, but in the short-term, there do not appear to be any drawbacks associated with the new procedure.

## Data Availability

The data that support the findings of this study are not openly available due to reasons of sensitivity and are available from the corresponding author upon reasonable request. Data are located in controlled access data storage at the U.S. Department of Veterans Affairs. Requests to access the datasets should be directed to ML, MaryGrace.Lauver@va.gov.

## Author disclaimer

This work was authored as part of the Contributor’s official duties as an Employee of the United States Government and is therefore a work of the United States Government. In accordance with 17 U.S.C. 105, no copyright protection is available for such works under U.S. Law. The views expressed in this paper are those of the authors and do not necessarily reflect the position or policy of the United States Department of Veterans Affairs (VA), Veterans Health Administration (VHA), or the United States Government.
